# Merging Heterocyclic Chemistry and Biocatalysis in One-Pot Processes through Compartmentalization of the Reaction Steps

**DOI:** 10.3390/bioengineering5030060

**Published:** 2018-08-01

**Authors:** Nadine Zumbrägel, Harald Gröger

**Affiliations:** Chair of Organic Chemistry I, Faculty of Chemistry, Bielefeld University, Universitätsstraße 25, 33615 Bielefeld, Germany; nadine.zumbraegel@uni-bielefeld.de

**Keywords:** Asinger-type multi-component reaction, asymmetric synthesis, biocatalysis, cascades, compartmentalization, heterocycles

## Abstract

A proof of concept for a one-pot process merging a heterocycle formation by a classical chemical approach at basic conditions with a biocatalytic reduction, running at neutral pH conditions, is reported. A crucial component for this process is the compartmentalization of the single reactions by the use of polydimethylsiloxane thimbles. This process was applied successfully towards an asymmetric synthesis of (*S*)-2,2,3-trimethyl-1-thia-4-azaspiro[4.4]nonane, leading to excellent enantioselectivities of 99% enantiomeric excess (*ee*).

## 1. Introduction

The development of chemoenzymatic one-pot processes for particularly industrially relevant compound classes by combining biotransformations with chemocatalytic or other chemical reactions has recently gained increasing interest since intermediate isolations can be avoided, thus offering an option for a higher resource efficiency of such multi-step syntheses by minimizing solvent consumption and overall waste formation [1]. It is noteworthy that engineering of such processes has also been achieved for combining enzyme catalysis and chemocatalytic reactions, being not compatible with each other [2]. Successful engineering concepts for merging non-compatible catalytic systems with each other include, for example, biphasic systems [3,4], supramolecular hosts [5], artificial metalloenzymes [6,7,8], or spatial separation by flow chemistry [4,9]. Recently, the separation of the biocatalytic from the chemocatalytic reaction by means of polydimethylsiloxane (PDMS)-thimbles turned out as another suitable strategy for this purpose. Originally pioneered by the Bowden group [10,11,12] for merging chemocatalytic and “classic” chemical reactions, the Gröger group demonstrated the usefulness of this concept also for combining non-compatible chemo- and biocatalytic transformations, exemplified for the combination of a Wacker-oxidation with an enzymatic reduction [13]. In the meantime, this methodology was applied to a range of other combinations of metal-catalyzed transformations (Suzuki reactions, Wacker oxidations) and biotransformations (e.g., halogenases, transaminases, amine dehydrogenases) [14,15,16].

In continuation with our recent studies on the chemoenzymatic enantioselective synthesis of sulfur-containing heterocyclic amines consisting of an initial formation of heterocyclic imines and subsequent enzymatic reduction [17], we became interested to combine these reactions within a one-pot cascade process. As the “process windows“ for these two reactions differ strongly from each other (e.g., in terms of required pH conditions), we envisioned that the compartmentalization concept using PDMS thimbles applied already successfully for a range of combinations of chemo- and biocatalytic transformations [10,11,12,13,14,15,16] might be an option for such a combination of heterocyclic chemistry and biocatalysis. In the following, we report our results in this field demonstrating a proof of concept for such an engineered process, which to the best of our knowledge represents the first example of a one-pot process merging a heterocycle formation through a classic chemical process at strongly basic conditions with a biocatalytic reaction running at neutral pH conditions. This process concept, which was successfully applied towards an asymmetric one-pot synthesis of (*S*)-2,2,3-trimethyl-1-thia-4-azaspiro[4.4]nonane (**4a**), also opens up an option for combining further reactions in the field of formation of heterocycles with enzymatic reactions towards one-pot processes running in water.

## 2. Materials and Methods 

### 2.1. Preparation of the PDMS Thimbles for Compartmentalization of the Reaction Steps

The PDMS thimbles were prepared according to Uthoff et al. [14]. The surface of a glass vial (1.5 mL) is passivated by addition of a few drops of trichloro-(1H, 1H, 2H, 2H-perfluoroctyl)silane onto the glass vial and incubation in a desiccator at 45 mbar over night. Sylgard^®^ 184 (Sigma-Aldrich, St. Louis, MO, USA) is mixed, degassed, and heated to 65 °C for 5 min. The glass vial is dipped into the solution of Sylgard^®^ 184 until no formation of new drops is observed and incubated upside down at 65 °C for 1 h. This step is repeated twice. In the third round the glass vial is incubated upright for 1 h at 120 °C to cure the PDMS. For release of the PDMS thimble from the glass vial, the glass vial is incubated in *n*-hexane. The PDMS thimble is washed with dichloromethane and water and afterwards used for reactions.

### 2.2. Construction and Expression of Whole-Cell Catalyst

The imine reductase [18], as well as the glucose dehydrogenase, used in this work are literature-known enzymes. *Escherichia coli* strain BL21(DE3), which was used for expression, and pACYCDuet-1 vector were purchased from Novagen (Madison, WI, USA). The whole-cell catalyst was constructed as a two-plasmid-system, harbouring the gene for the glucose dehydrogenase from *Bacillus subtilis* in a pACYCDuet-1 vector [17,19,20], and the gene for the imine reductase from *Mycobacterium smegmatis* in the commercially available pET-22b(+) vector [18]. A preculture of *E. coli* BL21(DE3) carrying the two recombinant plasmids was cultivated over-night at 37 °C in 10 mL LB medium, containing 80 µg·mL^−1^ of carbenicillin and 28 µg·mL^−1^ of chloramphenicol. The main culture containing 600 mL TB medium, 80 µg·mL^−1^ of carbenicillin, and 28 µg·mL^−1^ of chloramphenicol, was inoculated with the starting culture to a final concentration of 1%. At an OD600 between 0.4 and 0.6, the production of recombinant protein was induced by addition of isopropyl-thio-β-d-galactoside (IPTG) to a final concentration of 0.5 mM. Cultures were shaken at 25 °C for 20 h and cells harvested by centrifugation (4000 *g*, 30 min, 4 °C). For lyophilization of the cells, a 50% cell suspension in water was used and the resulting lyophilized cells were stored in a freezer at −20 °C.

### 2.3. GC Analytics for the Determination of the Conversion

Conversions for the biotransformations of 3-thiazoline (**3a**) to the corresponding 3-thiazolidine (**4a**) and overall conversions for the synthesis of **4a** as well as amount of diffusion of **3a** through the PDMS membrane were determined by means of gaschromatography. Analysis was carried out using the gaschromatograph system GC-2010 Plus (Shimadzu, Kyoto, Japan) equipped with ZB-5MSi column (Phenomenex, Torrance, CA, USA, 30 m × 0.25 mm × 0.25 µm; N_2_; linear velocity 46.9 cm·s^−1^ split mode 1:10; total flow 28.8 mL·min^−1^; purge flow 3.0 mL·min^−1^; column flow 2.34 mL·min^−1^; pressure 140.4 kPa) and coupled to an AOC-20i/s auto injector/auto sampler.

Temperature program: 40 °C–200 °C, 10 °C·min^−1^.
Retention time for 2,2,3-trimethyl-1-thia-4-azaspiro[4.4]non-3-ene (**3a**): 8.52 min.
Retention time for 2,2,3-Trimethyl-1-thia-4-azaspiro[4.4]nonane (**4a**): 8.96 min.

### 2.4. Derivatization of 3-Thiazolidine (**4a**) with Phenylisocyanate

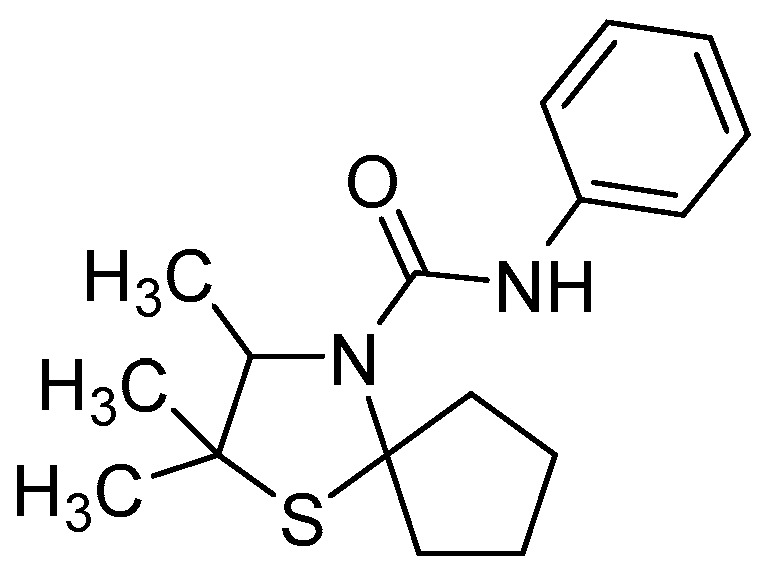



The derivatization is conducted according to Reiners et al. [21]. 2,2,3-Trimethyl-1-thia-4-azaspiro[4.4]nonane (**4a**) (54.1 mg, 0.29 mmol) is dissolved in diethylether (0.5 mL). Phenylisocyanate (33.5 µL, 0.31 mmol) and cyclohexane (0.25 mL) are added, the reaction mixture is stirred over night, and the solvent is evaporated meanwhile. The solid is dried in vacuo, yielding 2,2,3-Trimethyl-*N*-phenyl-1-thia-4-azaspiro[4.4]nonan-4-carboxamide (**5a**) (82.3 mg, 0.27 mmol, 93%) as white solid.

**^1^H-NMR** (500 MHz, CDCl_3_): δ (ppm) = 7.38–7.33 (m, 2 H, Ar-**H**), 7.32–7.27 (m, 2 H, Ar-**H**), 7.06–7.03 (m, 1 H, Ar-**H**), 3.88 (q, *J* = 6.40 Hz, 1 H, C3-**H**), 3.03–2.96 (m, 1 H, (C**H_2_**)**_4_**), 2.86–2.79 (m, 1 H, (C**H_2_**)**_4_**), 2.18–2.09 (m, 1 H, (C**H_2_**)**_4_**), 1.97–1.83 (m, 4 H, (C**H_2_**)**_4_**), 1.62–1.60 (m, 1 H, (C**H_2_**)**_4_**), 1.63 (s, 3 H, C2-C**H_3_**), 1.44 (d, *J* = 6.35 Hz, 3 H, C3-C**H_3_**), 1.32 (s, 3 H, C2-C**H_3_**).

**^13^C-NMR** (126 MHz, CDCl_3_): δ (ppm) = 152.4 (**C**=O), 138.9, 129.1, 123.4, 120.4 (Ar-**C**), 80.8 (**C5**), 68.0 (**C3**), 51.9 (**C2**), 41.7, 41.1, 32.1, 23.7 ((**C**H_2_)_4_), 25.1 (C2-**C**H_3_), 25.1 (C2-**C**H_3_), 17.5 (C3-**C**H_3_).

**HRMS (ESI)***m/z* calculated for C_17_H_25_N_2_OS [M + H]^+^: 305.16821, found: 305.1687.

### 2.5. HPLC Analytics for the Determination of the Enantiomeric Excess

For analysis of the enantiomeric excess of 3-thiazolidine **4a**, samples were derivatized according to Section 2.4. The corresponding solids were dissolved in dichloromethane after derivatization and analyzed by means of a LC2000 SFC-HPLC system from Jasco (Easton, Los Angeles, CA, USA) with HPLC column Chiralpak IC from Daicel (Tokyo, Japan) (supercritical CO_2_:EtOH (Et_2_NH) = 90:10 (0.01), 1 mL·min^−1^, 20 °C, 10 MPa, 210 nm). Enantiomeric excess was determined based on area% of the enantiomers.

In our previous work, the absolute configuration of (*S*)-**4a** was determined by vibrational circular dichroism (VCD) spectroscopy [17].

Retention times for enantiomers of **5a** (derivatized **4a**):(*S*)-**5a**:13.52 min, (*R*)-**5a**:17.57 min. 

### 2.6. Biotransformation of 3-Thiazoline (**3a**) in the Presence of Different Amounts of Dimethyl Sulfoxide (DMSO)/3-Chloro-3-methylbutan-2-one (**1**)/Cyclopentanone (**2**)

The biotransformations (10 mL) were performed starting from a 20 mM concentration of 2,2,3-trimethyl-1-thia-4-azaspiro[4.4]non-3-ene (**3a**), 1 mg·mL^−1^ of lyophilized whole-cell catalyst (prepared from 2 mg·mL^−1^ of cell suspension in 200 mM KPi buffer pH 7, construction of the whole-cell catalyst is described in Section 2.2), 40 mM of D-glucose, 0.1 mM of NADP^+^, and 10% *v*/*v* of methanol or 20% or 30% *v*/*v* of dimethyl sulfoxide (DMSO) as co-solvent in distilled water. Biotransformations in the presence of 3-chloro-3-methylbutan-2-one (**1**) (5 or 20 mM) or cyclopentanone (**2**) (5 or 20 mM) were performed at a co-solvent concentration of 20% *v*/*v* DMSO. The reactions were stirred at 30 °C for 18 h. The reaction was stopped by adding 1 mL of 32% NaOH solution and 10 mL of dichloromethane. Phase separation was promoted by centrifugation. The organic phase was dried over magnesium sulfate and the conversion was determined by analyzing the organic phase by means of achiral GC (2.3).

### 2.7. Studies on Diffusion of **3a** through the PDMS Membrane

For examination of the diffusion of **3a** through the PDMS membrane, 3-thiazoline (**3a**) (345 mg, 1.87 mmol) was added to aqueous ammonia solution (5.6 M, 675 µL) inside the thimble. DMSO (10 mL) was added to the outer compartment and both solutions were stirred (inside the thimble stirring with the mechanical stirrer, outside stirring was carried out using a stirring bar). After 0.75 h, 2 h, 3.5 h, 5 h, and 6 h a sample of DMSO (100 µL) from the outer compartment was transferred to a GC vial, mixed with dichloromethane (100 µL), and analyzed by means of achiral GC (2.3). After 6 h, the inner phase (inside the timble) was extracted with dichloromethane (3 times). The combined organic phases were dried over magnesium sulfate and the solvent was evaporated in vacuo. The remaining yellow oil was dissolved in dichloromethane and analyzed by means of achiral GC (2.3). The concentration of 3-thiazoline (**3a**) in the outer compartment was calculated by means of a standard curve and related to the maximum concentration of **3a** in the outer compartment.

### 2.8. Synthesis of 3-Thiazoline (**3a**) via Asinger-Reaction in Different Reactor Types

Finely crushed sodium hydrosulfide monohydrate (333 mg, 4.49 mmol) was mixed with cyclopentanone (**2**) (378 µL, 4.49 mmol) and aqueous ammonia-solution (675 µL, 13.3 M) inside the thimble or a glass vial. 3-Chloro-3-methylbutan-2-one (**1**) (542 mg, 4.49 mmol) was added at room temperature and the solution was stirred (stirring bar or mechanical stirrer) for 18 h at room temperature. The reaction solution was extracted with dichloromethane (3 times), the combined organic phases were dried over magnesium sulfate, and the solvent was evaporated in vacuo. The remaining yellow oil was dissolved in dichloromethane and analyzed by means of achiral GC (2.3). The conversion towards 3-thiazoline (**3a**) was calculated by means of a standard curve.

### 2.9. One-Pot Process Combining the Asinger-Synthesis of 3-Thiazoline (**3a**) with an Enzymatic Reduction via Compartmentalization of the Reaction Steps

Finely crushed sodium hydrosulfide monohydrate (333 mg, 4.49 mmol) was mixed with cyclopentanone (**2**) (378 µL, 4.49 mmol) and aqueous ammonia-solution (675 µL, 13.3 M) inside the timble. 3-Chloro-3-methylbutan-2-one (**1**) (542 mg, 4.49 mmol) was added and the solution was stirred (mechanical stirrer) for 18 h at room temperature. DMSO (10 mL) was added to the outer compartment and both solutions were stirred (inside the thimble stirring with the mechanical stirrer, outside stirring was carried out using a stirring bar) for 6 h. To the outer compartment, didestilled water (7.8 mL), 200 mM D-glucose (6.7 mL of a 1.5 M stock solution), 0.1 mM NADP^+^ (0.5 mL of 10 mM stock solution), and 8 mg·mL^−1^ lyophilized whole-cell catalyst (prepared from 16 mg·mL^−1^ of cell suspension in 200 mM KPi buffer pH 7, construction of the whole-cell catalyst is described in Section 2.2) were added. The outer reaction compartment is heated to 30 °C and both reaction solutions (inner and outer compartment) were stirred for 24 h. The reaction was stopped by adding 2 mL of 32% NaOH solution and 50 mL of dichloromethane to the outer reaction solution. Phase separation was promoted by centrifugation. The organic phase was dried over magnesium sulfate and the conversion was determined by analyzing the organic phase by means of achiral GC (2.3). The organic solvent was evaporated in vacuo and part of the product was derivatized according to Section 2.4 and then analyzed by chiral SFC-HPLC (2.5). 2,2,3-Trimethyl-1-thia-4-azaspiro[4.4]nonane (**4a**) (100 mg, 0.54 mmol, 12%) was isolated by flash column chromatography using the Biotage “Isolera One“ flash chromatography system (5–40% ethyl acetate in cyclohexane) as yellowish oil with an isolated yield of 12% and a purity of 97% (determined by ^1^H NMR spectroscopy). 

**^1^H-NMR** (500 MHz, CDCl_3_): δ (ppm) = 3.10 (q, *J* = 6.60 Hz, 1 H, C3-**H**), 2.13–2.05 (m, 1 H, (C**H_2_**)**_4_**), 1.95–1.78 (m, 2 H, (C**H_2_**)**_4_**), 1.77–1.63 (m, 5 H, (C**H_2_**)**_4_**), 1.41 (s, 3 H, C2-C**H_3_**), 1.19 (s, 3 H, C2-C**H_3_**), 1.09 (d, *J* = 6.61 Hz, 3 H, C3-C**H_3_**).

**^13^C-NMR** (126 MHz, CDCl_3_): δ (ppm) = 81.5 (**C**5), 67.1 (**C**3), 59.9 (**C**2), 44.8, 42.4, 24.4, 23.9 ((**C**H_2_)**_4_**), 27.8 (C2-**C**H_3_), 26.1 (C2-**C**H_3_), 13.7 (C3-**C**H_3_).

The analytical data corresponds with literature data [17,21].

## 3. Results and Discussion

### 3.1. Compartmentation Approach

**Figure 1:**
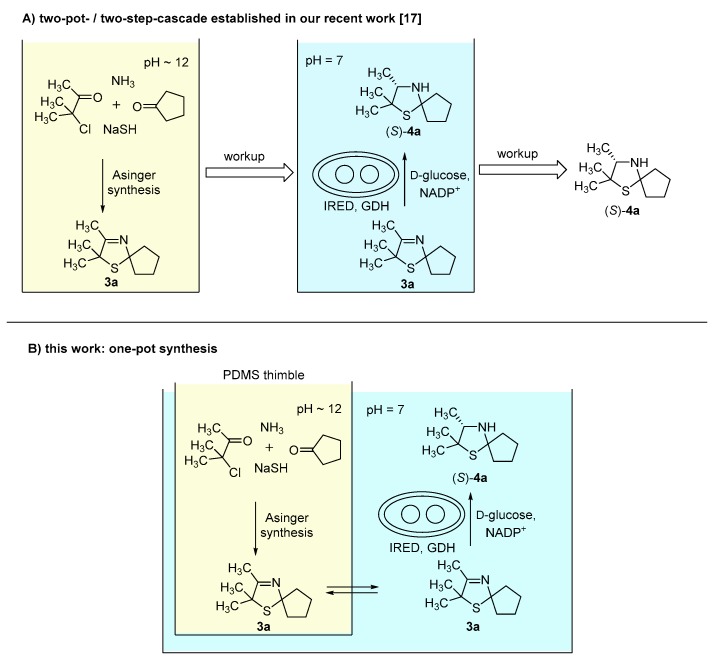
(**A**) Concept of our recent work: Synthesis of 3-thiazolidines (**4a**) in a two-pot-/two-step cascade [17]. (**B**) Concept of this work: One-pot synthesis towards 3-thiazolidines (**4a**) via compartmentalization approach using polydimethylsiloxane (PDMS) thimbles.

In our recent study [17], synthesis of 3-thiazolidines (**4**) was performed via a two-pot-/two-step-cascade (Figure 1A). The first step consists of an Asinger-type multi-component reaction as an efficient synthesis of 3-thiazolines (**3**), which requires a basic pH around 12. The second step is an enzymatic reduction of 3-thiazolines (**3**) catalyzed by imine reductases (IREDs), which proceeds at a pH optimum of 7. Due to their different required pH conditions, the single reaction steps are not compatible with each other. Therefore, we chose a compartmentalization approach to develop a one-pot synthesis for the asymmetric synthesis of 3-thiazolidines (**4**). We used polydimethylsiloxane (PDMS) thimbles, which in earlier works were shown to be permeable for hydrophobic and (mostly) non-permeable for water-soluble components [10,11,12,13,14]. Thus, the use of PDMS thimbles was chosen for compartmentalization of Asinger-type reaction and IRED-catalyzed reduction. Sodium hydrosulfide and biocatalyst remain in their compartment, whereas 3-thiazoline (**3**) can pass the membrane after being formed in the Asinger-synthesis to serve as substrate in the second reaction step (Figure 1B).

### 3.2. Studies on Asinger-Type Multi-Component Reaction

**Figure 2:**
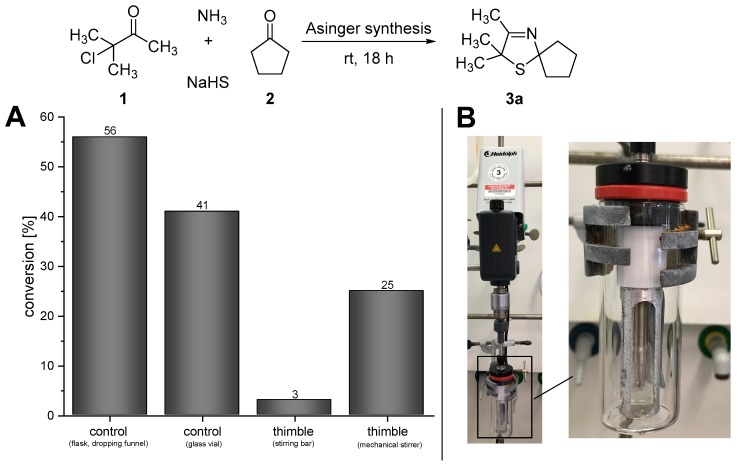
(**A**) Results for the Asinger-type multi-component reaction under different conditions. (**B**) Mechanical stirrer based on a KPG stirrer for high stirring speed inside the PDMS thimble.

In order to design this one-pot process for the synthesis of 3-thiazolidines (**4a**), at first we studied the initial reaction step of the cascade, namely the Asinger-type multi-component reaction. Since the Asinger-type synthesis is conducted within a two-phase system of aqueous and organic phase, the stirring rate and scale of the reaction turned out to have a tremendous effect on the conversion. When the Asinger-type synthesis was performed in a flask on a 200 mmol scale with stepwise addition of the α-chlorinated ketone under cooling in the presence of dichloromethane (as a “benchmark experiment”) [17], a conversion of 56% of 2,2,3-trimethyl-1-thia-4-aza-spiro[4.4]non-3-ene (**3a**) was obtained (Figure 2A, control (flask, dropping funnel)). However, when the Asinger-synthesis was performed on smaller scale (4.5 mmol) in a glass vial at room temperature (for comparison with the subsequently described experiments with the PDMS thimble, which could not be cooled), allowing a high stirring rate, conversion decreased to 41% after 18 h of reaction time at room temperature (Figure 2A, control (glass vial)). Since the PDMS thimbles are sensitive to shearing forces, which increase with higher stirring rates of the stirring bar inside the thimbles, the initial Asinger-type reaction, being conducted inside the thimble at room temperature, was performed with a low stirring rate using a stirring bar. In this case, only very low conversion of 3% for 3-thiazoline (**3a**) was obtained after 18 hours of reaction time (Figure 2A, thimble (stirring bar)). As such a low conversion can be directly linked to an unsufficient mixing due to a low stirring rate, we developed a mechanical stirrer based on an overhead stirrer (KPG stirrer) to enable high rates and circumvent high shearing forces at the same time (Figure 2B). The use of this mechanical stirrer increased the conversion in the Asinger synthesis significantly to 25% after 18 h (Figure 2A, timble (mechanical stirrer)), representing a promising starting point for an application in the cascade reaction towards the desired one-pot synthesis of (*S*)-2,2,3-trimethyl-1-thia-4-azaspiro[4.4]nonane (**4a**).

### 3.3. Diffusion Efficiency and Biocatalytic Reduction Using Imine Reductases

**Figure 3:**
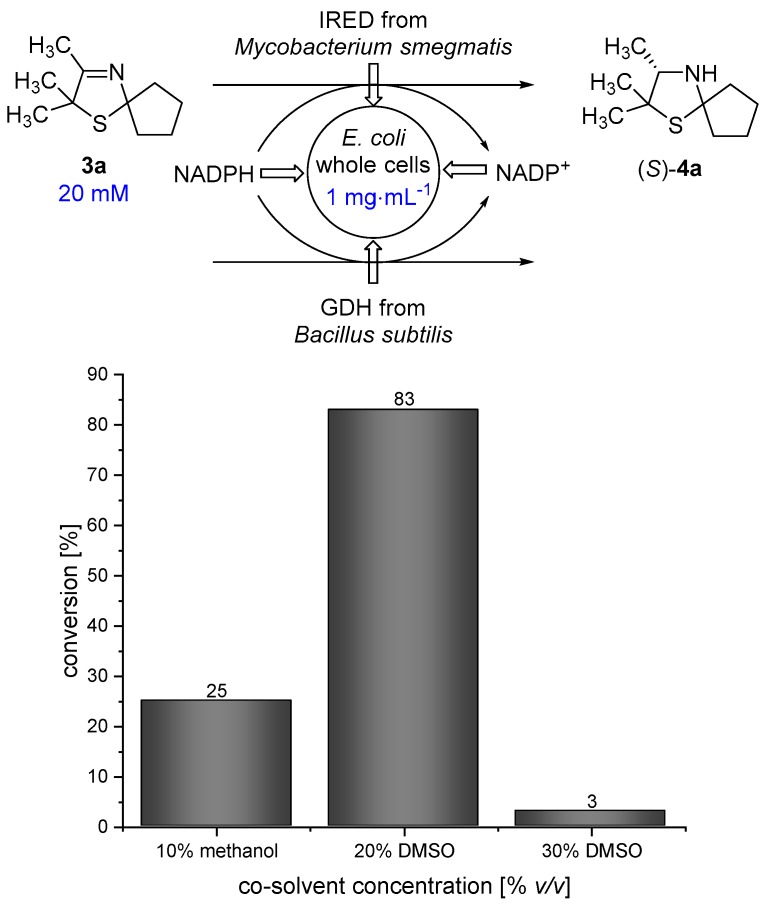
Results for the reduction of 3-thiazoline (**3a**) using an *E. coli* whole-cell catalyst in the presence of different amounts of methanol/dimethylsulfoxide (DMSO) as a co-solvent.

When performing a one-pot synthesis of 3-thiazolidine (**4a**) using PDMS thimbles for compartmentalization, the diffusion rate of the 3-thiazoline (**3a**) is a critical point that needs to be examined. The presence of an organic solvent turned out to be beneficial for the diffusion of **3a** through the PDMS membrane according to the results of Uthoff et al. [14]. Therefore, we were interested in using a water-miscible organic solvent being tolerated by the imine reductase to enhance the diffusion of **3a**. Methanol and dimethylsulfoxide (DMSO) represent such suitable co-solvents for the biocatalytic reduction using a whole-cell catalyst. In order to obtain a high diffusion rate of 2,2,3-trimethyl-1-thia-4-azaspiro[4.4]non-3-ene (**3a**), a high co-solvent concentration in the outer aqueous compartment is desirable. We performed biotransformations of 3-thiazoline (**3a**) in the presence of different amounts of methanol and DMSO at 30 °C for 18 h in order to examine the maximum amount of co-solvent that is tolerated by the *Escherichia coli* (*E. coli*) whole-cell catalyst. In analogy to our previous work [17] an *E. coli* whole-cell catalyst was used, overexpressing the imine reductase from *Mycobacterium smegmatis* [18] and a glucose dehydrogenase (GDH) from *Bacillus subtilis* for in situ-cofactor recycling. The genes, encoding for IRED and GDH, have been inserted on different plasmids. Unfortunately, the conversion of 3-thiazoline (**3a**) towards **4a** is quite low, with only 25% in the presence of 10% *v*/*v* methanol. Therefore, methanol does not represent a suitable co-solvent that can be used in high concentrations which are required for the envisaged process. We found that the reaction proceeds smoothly in the presence of 20% *v*/*v* DMSO giving 83% conversion after 18 h of reaction time. In contrast, the conversion is dramatically decreased to 3% in the presence of 30% *v*/*v* DMSO. Thus, as a compromise between high co-solvent concentration and high conversion, 20% *v*/*v* DMSO was chosen for the cascade reaction (Figure 3). 

With the optimal co-solvent concentration for the cascade reaction in hand, we examined the diffusion of **3a** through the PDMS membrane using an aqueous 20% *v*/*v* DMSO solution in the outer compartment. Unfortunately, we found that the diffusion of **3a** was slow with only 24% in the outer media after 22 h. Therefore, we studied the diffusion using pure DMSO in the outer compartment and observed that diffusion of **3a** proceeds faster yielding 60% of **3a** in the DMSO phase after 6 h.

**Figure 4:**
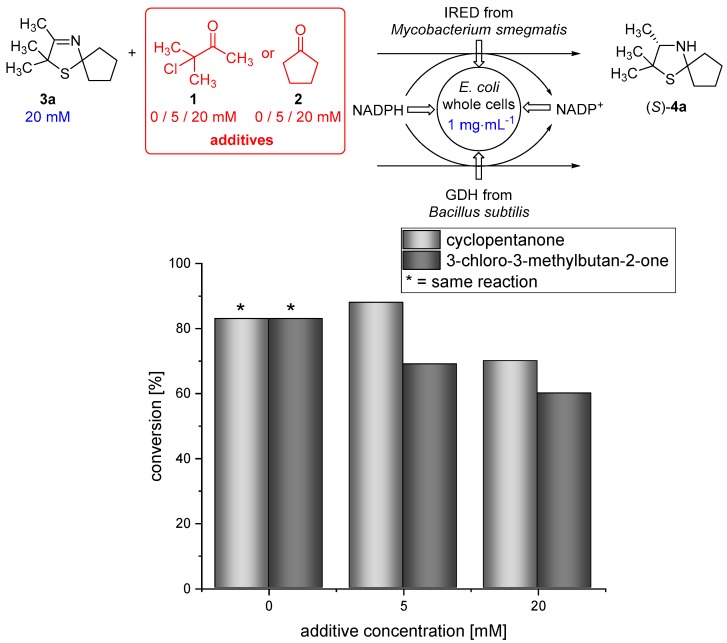
Results for the biotransformation of 3-thiazoline (**3a**) in the presence of different concentrations of either cyclopentanone (**2**) or 3-chloro-3-methylbutan-2-one (**1**).

Moreover, 3-chloro-3-methylbutan-2-one (**1**) and cyclopentanone (**2**), which are used as substrates in the Asinger-type multi-component reaction (Figure 1 and Figure 2), are able to pass the membrane (data not shown). Therefore, we examined if the biocatalytic reduction is compatible with the α-chlorinated ketone **1** and cyclopentanone (**2**). Thus, biotransformations of 3-thiazoline (**3a**) in the presence of different concentrations (5 and 20 mM) of either cyclopentanone (**2**) or 3-chloro-3-methylbutan-2-one (**1**) were conducted at 30 °C for 18 h. We were pleased to find that the IRED-catalyzed reduction proceeds smoothly, even in the presence of 5 or 20 mM of **1** or **2**, still giving 70% conversion in the presence of 20 mM cyclopentanone (**2**) and 60% conversion when the biotransformation was conducted in the presence of 20 mM 3-chloro-3-methylbutan-2-one (**1**). This represents a slight decrease in conversion compared to the biotransformation under standard conditions (Figure 4), being acceptable for the cascade reaction.

### 3.4. One-Pot Process Combining the Asinger-Synthesis of 3-Thiazoline (**3a**) with an Enzymatic Reduction via Compartmentalization of the Reaction Steps

**Figure 5:**
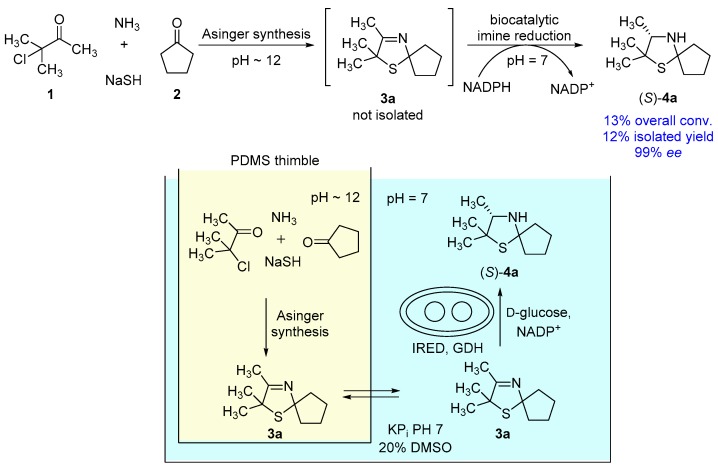
One-pot process towards synthesis of (S)-2,2,3-trimethyl-1-thia-4-azaspiro[4.4]nonane (**4a**).

With the optimized single reaction steps for the synthesis of (*S*)-2,2,3-trimethyl-1-thia-4-azaspiro[4.4]nonane (**4a**) in hand, we performed the envisaged one-pot process (Figure 5). The process was carried out in a sequential fashion, since pure DMSO is required for a high diffusion rate of 3-thiazoline (**3a**) through the PDMS membrane. Consequently, at first the Asinger-type multi-component reaction is conducted inside the timble to synthesize **3a** for 18 h at room temperature, followed by diffusion of the 3-thiazoline (**3a**) for 6 h using pure DMSO in the outer compartment. As the next step, buffer, NADP^+^, D-glucose, and lyophilized whole-cell catalyst are added to obtain an aqueous solution for the biotransformation containing 20% *v*/*v* DMSO. After a reaction time of 24 h at 30 °C for the biotransformation, we found that 2,2,3-trimethyl-1-thia-4-azaspiro[4.4]nonane (**4a**) was formed with an overall conversion of 13% and an excellent enantioselectivity of 99% ee for the (*S*)-enantiomer. After work-up, the desired 3-thiazolidine(*S*)-**4a** was isolated in 12% yield (Figure 5). Even though an overall conversion of 13% is relatively low, this overall conversion fits well with the expected conversion being calculated from the single reaction steps. In the one-pot process, we observed a quantitative conversion in the biotransformation step after extraction of the aqueous phase with dichloromethane. Therefore, the biotransformation is not representing a limiting step in the one-pot process. Moreover, the expected conversion for the first two reaction steps, consisting of Asinger-synthesis and diffusion of **3a** through the PDMS membrane is 15% (taking into account 25% for the Asinger-synthesis and 60% for the diffusion). Accordingly, the observed overall conversion of 13% in the one-pot process is in good agreement with the expected conversion.

## 4. Conclusions

We reported the first proof of concept for a one-pot process merging a heterocycle formation through a classic chemical process at strongly basic conditions with a biocatalytic reaction under neutral pH conditions. For compartmentalization of the incompatible reaction step, PDMS thimbles were used. This one-pot process was applied successfully towards a highly enantioselective synthesis of (*S*)-2,2,3-trimethyl-1-thia-4-azaspiro[4.4]nonane utilizing commercially available and cheap starting materials.
